# The Institutional Worker

**Published:** 1912-04-27

**Authors:** 


THE
### INSTITUTIONAL WORKER
Being a Special Supplement to "The Hospital"

### Editor's Notices.

**Contributions:**

Contributions should be written, or preferably typed, on one side of the paper only, and all articles sent in are accepted upon the distinct understanding that they are forwarded to THE HOSPITAL only.

The Editor cannot undertake to return MSS. not used, but when a stamped directed envelope is enclosed the MSS. may be returned if a special request is made.

Accepted articles and paragraphs of news will be paid for after publication at the scale rate.

**Address:**

To prevent delay all contributions and letters on editorial business must be addressed exclusively to the Editor, "The Hospital," 29 Southampton Street, Strand, London, W.C. It is important that this regulation shall be strictly observed.

**Correspondence:**

Correspondence on all subjects is invited. The name and address of correspondents must be given as a guarantee of good faith, but not necessarily for publication.

**Special Articles:**

Special articles are invited, and questions, inquiries, and paragraphs upon all matters relating to the work, administration, and management of general, special, mental, fever, cottage and convalescent hospitals, sanatoria, homes, institutions, societies and organisations for the treatment or care of the sick, injured and dependants of all classes. Every matter affecting the interests and welfare of the staff of all grades working in these institutions will receive special consideration.

**Administrative Medicine, Research and Items of News:**

Special terms will be paid for approved articles on subjects in this wide field by experts who have made some section of it their special study and interest. We wish to give prominence to every movement tending to advance accuracy of diagnosis, efficiency in treatment, and the development of modern methods to eradicate disease and promote the welfare of the suffering.

**A Bureau of Information:**

We invite inquiries and applications for information and help in providing for that numerous class of sufferers who require special care or house-room which they cannot obtain in their own homes or secure for themselves. We cannot, however, prescribe, or recommend practitioners.

**Books for Review:**

Publishers are particularly requested to send advance proofs of any new books of importance whenever possible, as the Editor has made arrangements to publish immediate reviews on a new plan.

**Photographs, Plans, Blocks, and Illustrations:**

It is requested that wherever possible MSS. may be accompanied by illustrations in any of the above forms. The name of the sender and of the article to which it belongs should be written on the back of each photograph, drawing, or block for purposes of identification.

**Local Papers:**

Newspapers containing reports or news paragraphs should be marked and addressed to the Sub-Editor.

**The Coupon System:**

A coupon will be found at the bottom of the third inside page of the cover each week. This coupon must be attached to every question or inquiry to which an answer is desired in THE HOSPITAL.

### Our Advertisement Pages.

NOT the least of the many interesting and unique features of this week's issue of THE HOSPITAL is the large amount of useful information contained in the advertisement pages. Here will be found the results of commercial enterprise in its efforts to secure the most efficient appliances for the economical management of every department of institutional work. Consequently, the particulars contained in these pages cannot fail to prove of the utmost assistance to every keen and progressive institutional official. It is our purpose to make the pages of THE HOSPITAL as interesting and as comprehensive as possible, so that it is *the journal* of all that is essential and up to date in hospital and institutional affairs. In these circumstances, we would submit that our readers will find it to their interest to give some time and attention to our advertisement pages.

### Manager's Notices.

**All letters on business matters must be addressed to The Manager, "The Hospital," The Hospital Building, 28 and 29 Southampton Street, London, W.C.**

**Subscriptions,** which may commence at any time, are payable in advance. Cheques and Post-Office orders should be crossed London County and Westminster Bank, Covent Garden branch, and made payable to the Manager of THE HOSPITAL.

RATES OF SUBSCRIPTION (payable in advance).

UNITED KINGDOM.

| | | |
|---|---|---|
| Three Months (including Postage) | ... ... ... ... ... | 2s. 0d. |
| Six Months do. | ... ... ... ... ... | 4s. 0d. |
| Twelve Months do. | ... ... ... ... ... | 6s. 6d. |

FOREIGN AND COLONIAL.

| | | |
|---|---|---|
| Three Months (including Postage) | ... ... ... ... ... | 2s. 6d. |
| Six Months do. | ... ... ... ... ... | 5s. 0d. |
| Twelve Months do. | ... ... ... ... ... | 8s. 8d. |

**Advertisements:**

To insure insertion, all advertisements for THE HOSPITAL must reach the Manager not later than Wednesday morning in each week. For scale of charges see pages vii and 2.

**Special Rates** will be quoted for those institutions that agree to send all their public notices to THE HOSPITAL each year, so as to make it a file of such announcements for ready reference.

This form may be used for small prepaid advertisements, and when filled up should be posted to

*The Manager*, THE HOSPITAL,
28 and 29 Southampton Street,
Strand, W.C.

Appointments Wanted, 21 words ... 1s. 0d.
Appointments Vacant, 21 words ... 2s. 6d.

### OFFICIAL APPOINTMENTS VACANT.

### Poor-Law Vacancies.

Assistant Clerk (£50), Holborn Union. Mr. J. A. Battersby, C. to G., Clerkenwell Road, E.C. April 29.

Assistant Cook (£22), Hunslet Union. Mr. F. W. Mee, C. to G., Hunslet, Leeds. May 1.

Assistant Laundry Superintendent (£24), Hammersmith Guardians. The C. to G., 206 Goldhawk Road, Shepherd's Bush, W. April 29.

Assistant Matron (£25), Wellingborough Union. Mr. T. J. Morgan, C. to G., Wellingborough. April 30.

Assistant Nurse (£25), Hexham Union. Mr. J. H. Nicholson, C. to G., Hexham. April 29.

Assistant Nurse (£26), Knaresborough Union. Mr. J. Smith, C. to G., Knaresborough. May 1.

Assistant Nursery Attendant (£21 18s.), St. George-in-the-East Guardians. Mr. R. M. Lochner, C. to G., Raine Street, Old Gravel Lane, E. April 30.

Assistant Nurse (£25), Bedford Union. Mr. W. Payne, C. to G., Bedford. April 29.

Assistant Nurse (£25), Sudbury Union. Mr. G. L. Andrewes, C. to G., Sudbury, Suffolk. April 29.

Assistant Nurse (£25), Dorking Union. Mr. W. James, C. to G., Dorking. April 30.

Assistant Nurse (£25), Wisbech Union. T. H. Stockdale, C. to G., 2 Ely Place, Wisbech. April 27.

Baker (£30), Faversham Union. Mr. G. Tassell, C. to G., Faversham. April 29.

Bathman, etc. (£30), Camberwell Guardians. Mr. C. S. Stevens, C. to G., Peckham Road, S.E. May 2.

Boys' Caretaker and Indoor Labour Master (£30), Macclesfield Union. Mr. F. May, C. to G., Macclesfield. April 29.

Charge and Midwifery Nurse (£32), Northampton Union. Mr. W. Fawkes, C. to G., Northampton. May 2.

Charge Nurse (£30), Bucklow Union. The C. to G., Knutsford. April 29.

Charge Nurse (£30), Canterbury Guardians. Mr. J. Plummer, C. to G., Canterbury. April 29.

Charge Nurses (2) (£35), Bedford Union. Mr. W. Payne, C. to G., Bedford. April 29.

Cook (£20), Romsey Union. Mr. A. J. Hartington, C. to G., Romsey. April 27.

Cook (£23), Hemel Hempstead Union. Mr. L. Smeathman, C. to G., Hemel Hempstead. April 29.

Cook and Assistant Matron (£25), Spalding Union. Mr. H. S. Maples, C. to G., Spalding. May 2.

Female Assistant Relieving Officer (£110), Camberwell Guardians. Mr. C. S. Stevens, C. to G., Peckham Road, S.E. May 1.

Female Attendant (12s. weekly), Bermondsey Guardians. Mr. E. Pitts Fenton, C. to G., 283 Tooley Street, S.E. May 6.

Female Industrial Trainer (£20), Spalding Union. Mr. H. S. Maples, C. to G., Spalding. May 2.

Female Teacher (£55), Southampton Guardians. Mr. A. J. Walden, C. to G., Southampton. May 4.

Foster-Mother (£25), Ecclesall Bierlow Union. Mr. J. E. Moulding, C. to G., The Edge, Sheffield. April 30.

General Assistant and Laundress (£20), Shifnal Union. Mr. H. R. Philips, C. to G., Shifnal. April 27.

Head Female Imbecile Attendant (£31), West Ham Union. T. Smith, C. to G., Union Road, Leytonstone. May 1.

Head Schoolmistress (£70), Walsall and West Bromwich School District. Mr. A. H. Ward, Clerk to the Managers, West Bromwich. April 27.

Industrial Trainer (£20), Shepton Mallet Union. Mr. A. E. Nalder, C. to G., Shepton Mallet. May 2.

Industrial Trainer and Assistant Matron (£20), St. Ives Union. Mr. G. D. Day, C. to G., St. Ives, Hunts. April 29.

Male Attendant (£30), Isle of Wight Union. Mr. A. G. Harrison, C. to G., Newport, I.W. April 30.

Male Baker and Cook (£30), Canterbury Guardians. Mr. J. Plummer, C. to G., Canterbury. April 29.

Master and Matron (£120 and £60), Hammersmith Guardians. The C. to G., 206 Goldhawk Road, Shepherd's Bush, W. May 2.

Master's Clerk (£25), Bucklow Union. The C. to G., Knutsford. April 29.

Nurses (£30), Winchester Union. F. Faithfull, C. to G., 105 High Street, Winchester. May 18.

Probationer Nurses (£10), Ecclesall Bierlow Union. J. E. Moulding, C. to G., Union Offices, The Edge, Sheffield. April 30.

Probationer Nurse (£10), Tonbridge Union. N. R. Stone, C. to G., 23 Church Road, Tunbridge Wells. May 2.

Probationers (£8), Wrexham Union. Mr. J. B. Bury, C. to G., Wrexham. April 30.

Relieving Officer, etc. (£135, commission and fees), Bromley Union. Mr. E. Haslehurst, C. to G., Bromley, Kent. May 2.

Sister (£35), Darlington Union. Mr. C. H. Leach, C. to G., Darlington. May 1.

Superintendent and Matron of Children's Home (£90 and £60), Ipswich Guardians. Mr. L. W. Greenhalgh, C. to G., Ipswich. May 8.

Superintendent Nurse (£45), Barton-upon-Irwell Union. Mr. J. W. Whitworth, C. to G., Patricroft, Manchester. April 30.

Superintendents of Children's Homes (married couple) (£40 and £30), Southampton Guardians. Mr. A. J. Walden, C. to G., Southampton. April 27.

Third Assistant Clerk (£100), Stoke-on-Trent Union. Mr. T. Wood, C. to G., Stoke-on-Trent. May 1.

Ward Sister (£30), Uxbridge Union. C. Woodbridge, C. to G., 38 High Street, Uxbridge. April 27.

### Institutional and Administrative Vacancies.

Matron (£45), Alnwick Infirmary. W. Hindmarsh, Hon. Secretary, 32 Bondgate Without, Alnwick. April 30.

Matron (£50), Yeovil District Hospital. F. W. Mayo, Hon. Sec., Swallowcliffe, Yeovil. May 10.

Matron (£90), Herefordshire General Hospital. W. A. W. Price, Secretary. May 6.

### Municipal Vacancies.

Assistant Inspector of Nuisances, etc. (£90), Gloucester T.C. Mr. G. S. Blakeway, Town Clerk, Gloucester. April 29.

Chief Inspector of Nuisances (£250), Hull T.C. Mr. H. A. Learoyd, Town Clerk, Hull. May 1.

Clerk to Epping Union, R.D.C., and U.A.C. (£260). Mr. J. C. Archer, Acting C. to G., Epping. May 1.

Clerk of Works (Baths) (50s. weekly), Nelson T.C. Mr. J. H. Baldwick, Town Clerk, Nelson. April 30.

Foreman of Sewage Works (52s. weekly), Bolton T.C. Mr. S. Parker, Town Clerk, Bolton. April 29.

Inspectors of Nuisances (2) (£110 each), Sedgefield R.D.C. Mr. J. W. Lodge, Clerk to the Council, Sedgefield, Ferryhill. April 29.

Inspector of Nuisances and Highway Surveyor (£120), Fordingbridge R.D.C. Mr. H. G. Beach, Clerk to the Council, Fordingbridge, Hants. May 3.

Junior Clerk (£40), Finsbury B.C. Mr. G. W. Preston, Town Clerk, Rosebery Avenue, E.C. April 29.

Lady Health Visitor (£75), City of Wakefield. W. W. Greenhalgh, Town Hall, Wakefield. May 6.

Matron (£65), Corporation of Sunderland Borough Sanatorium. Medical Office of Health, Town Hall, Sunderland. April 30.

Matron (£60), Borough of Crewe Isolation Hospital. A. B. McMaster, M.O.H., Municipal Buildings, Crewe. April 27.

Night Sister (£30), Tolworth Joint Hospital Board. Matron of the Hospital, Surbiton. April 29.

Rate Collectors (2) (£175), Hendon U.D.C. Mr. H. Humphris, Clerk to the Council, Hendon, N.W. April 29.

Sanitary Inspector (£150), Southwark B.C. Mr. P. H. Gray, Town Clerk, Walworth Road, S.E. April 29.

Surveyor's Assistant (25s. weekly), Earby U.D.C. Mr. J. E. Aldersley, Surveyor, Council Offices, Earby. May 3.

Surveyor's Junior Assistant (£65), Stoke-on-Trent T.C. Mr. A. Burton, Borough Surveyor, Stoke-on-Trent. May 1.

Tramway Repairs Foreman (£3 weekly), Newport T.C. Mr. H. Trewelling, Borough Engineer, Newport, Mon. April 30.

### Miscellaneous Vacancies.

School Nurse (£75), Rotherham Education Committee. J. A. Mair, Secretary, Education Offices, Rotherham. April 30.

School Nurse (£80), Reigate Education Committee. A. Lund Newell, B.A., Secretary, Municipal Buildings, Reigate. April 27.

